# Ultrasound‐microbubble cavitation facilitates adeno‐associated virus mediated cochlear gene transfection across the round‐window membrane

**DOI:** 10.1002/btm2.10189

**Published:** 2020-10-03

**Authors:** Zhen Zhang, Zhengnong Chen, Liqiang Fan, Thomas Landry, Jeremy Brown, Zhiping Yu, Shankai Yin, Jian Wang

**Affiliations:** ^1^ Otolaryngology Research Institute, 6th Affiliated Hospital Jiao Tong University Shanghai China; ^2^ Shanghai Key Laboratory of Sleep Disordered Breathing, 6th Affiliated Hospital, Jiao Tong University Shanghai China; ^3^ School of Biomedical Engineering Dalhousie University Halifax Canada; ^4^ School of Communication Science and Disorders Dalhousie University Halifax Canada

**Keywords:** cochlea, cochlear gene therapy, gene delivery, Guinea pigs, recombinant adeno‐associated virus, round window membrane, ultrasonic microbubbles cavitation

## Abstract

The round window of the cochlea provides an ideal route for delivering medicines and gene therapy reagents that can cross the round window membrane (RWM) into the inner ear. Recombinant adeno‐associated viruses (rAAVs) have several advantages and are recommended as viral vectors for gene transfection. However, rAAVs cannot cross an intact RWM. Consequently, ultrasound‐mediated microbubble (USMB) cavitation is potentially useful, because it can sonoporate the cell membranes, and increase their permeability to large molecules. The use of USMB cavitation for drug delivery across the RWM has been tested in a few animal studies but has not been used in the context of AAV‐mediated gene transfection. The currently available large size of the ultrasound probe appears to be a limiting factor in the application of this method to the RWM. In this study, we used home‐made ultrasound probe with a decreased diameter to 1.5 mm, which enabled the easy positioning of the probe close to the RWM. In guinea pigs, we used this probe to determine that (1) USMB cavitation caused limited damage to the outer surface layer or the RWM, (2) an eGFP‐gene carrying rAAV could effectively pass the USMB‐treated RWM and reliably transfect cochlear cells, and (3) the hearing function of the cochlea remained unchanged. Our results suggest that USMB cavitation of the RWM is a good method for rAAV‐mediated cochlear gene transfection with clear potential for clinical translation. We additionally discuss several advantages of the small probe size.

## Graphical abstract

A small ultrasound probe was developed for the treatment of round window membrane (RWM) in combination with the use of microbubble. The treatment causes a controllable damage on RWM that allows adeno‐associated virus (AAV) carrying genes to pass. The focused damage on the RWM can heal automatically without cause hearing loss. The new probe makes it possible to put through human eardrum across a hole that can be healed. Therefore, this method can be translated into human trials.

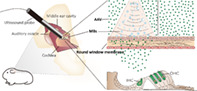

## 1 INTRODUCTION

Gene transfection is a critical procedure in both genetic studies and gene therapy. Gene transfection methods can be divided into two categories: non‐viral and viral. Viral methods of gene transfection are more efficient, despite recent rapid progress in non‐viral gene transfection methods.^1^, ^2^, ^3^, ^4^, ^5^, ^6^, ^7^, ^8^, ^9^, ^10^ Among what have been tested, recombinant adeno‐associated viruses (rAAV) exhibit clear advantages such as low immunogenicity, long‐lasting transfected gene expression in various host cells, and non‐exogenous DNA insertion into the genomes of transfected cells.^3^ This viral vector has been used in the gene therapy studies of auditory system in animal models^11^, ^12^, ^13^, ^14^, ^15^ and human trials.^11^, ^16^

The inner ear is highly isolated from surrounding organs and tissues. This unique feature makes it an ideal organ for genetic manipulation, with a low risk of side effects. However, this feature also makes it difficult to access. Generally, rAAV vectors must be injected into the inner ear, either via the round window membrane (RWM) or by cochleostomy. However, the injection disrupts the integrity of the inner ear, and might impair the hearing function.

The RWM has been explored as an approach to deliver drugs to the inner ears.^17^, ^18^ Unfortunately, the intact RWM is not permeable to rAAVs,^19^ and therefore rAAV‐mediated gene transfection via the RWM requires an injection.^16^, ^20^, ^21^, ^22^ This barrier could be overcome by increasing the RWM permeability temporarily. In one of our previous studies, we reported that this could be realized by treating the RWM with digestive enzymes.^23^, ^24^ Consequently, temporary RWM damage allows the rAAV to diffuse across the RWM. Since the treatment itself does not cause hearing loss, this method has a potential in cochlear gene therapy for the purpose of protection. However, the effectiveness of this treatment varied among individual subjects, likely due to variations in the RWM thickness and local tissue reactions to the enzyme solution.

Ultrasound‐mediated microbubble (USMB) cavitation can create small pores on the cell membrane (sonoporation).^25^, ^26^, ^27^ This temporary injury significantly increases the permeability of the cell membrane to large molecules.^28^, ^29^ The wound created by the USMB cavitation is self‐healable,^30^ and therefore such treatments do not permanently impair the normal functions of the treated cells. In addition to medication delivery,^26^, ^31^ the use of USMB cavitation for gene transfection via plasmid DNA, siRNA, and miRNA has been investigated.^27^, ^28^, ^32^ USMB‐mediated AAV gene transfection in the rat retina has also been reported.^33^ In that application, however, AAV was injected into the subretinal space before USMB was applied. Such an approach is not safe if applied in cochlear gene transfection.

Two studies have applied the USMB method for drug delivery via the RWM.^29^, ^34^ USMB effectively increased the permeability of the guinea pig RWM to large molecules such as biotin‐FITC.^29^ This method successfully facilitated the delivery of dexamethasone across the RWM and protected the cochleae against noise damage.^34^ However, the US probes used in these studies had a diameter of 6 mm. Such a large probe could not be inserted near the RWM even in human's ears. The long working distance needs a larger amount of working solution and a higher acoustic power, which may be potentially harmful.

In addition, no previous study has investigated the usefulness of this method for AAV‐mediated gene transfection via the RWM. As viral vectors are highly expensive, MB‐vector packaging or co‐administration of virus with MB appears to be impractical. In addition, the packing of vectors into MBs may deteriorate the activity of the virus. Therefore, the viral vector must be administered after USMB was applied to the RWM. This required that the wound would not be sealed quickly. Up to date, there is no data whether the damage by USMB will last. In one study, the sonoporation created by a single shot of USMB was healed in seconds. In other study, RWM damage by USMB was observed with electron microscopy with information how long the wound will be recovered.^35^

In this study, we developed a new ultrasound probe with a considerably smaller diameter (1.5 mm). By using this small probe, we were able to create intense, focalized damage to the RWM with a lower ultrasound power and a smaller amount of MB solution. The damage was limited to the outer epithelial layer of the RWM and lasted for more than a day. Effective eGFP gene transfection was observed when rAAV‐eGFP was administered after USMB treatment. Additionally, a new‐generation rAAV vector (AAV2/Anc80L65) was used to get satisfactory transfection.^36^, ^37^ This approach should be useful for the future development of cochlear gene therapies and the translation to humans.

## 2 MATERIALS AND METHODS

### 2.1 Animals and research design

Twenty‐seven 2‐month‐old male guinea pigs (albino Hartley) were obtained for this experiment from Shanghai Songlian Lab Animal Field (Shanghai, China) with body weight between 250 and 350 g. All animals passed Preyer's reflex test, an otoscope inspection and a baseline hearing evaluation with an auditory brainstem response (ABR) test. The guinea pigs were then randomly assigned into six different groups for (1) RWM structural changes observed at three time points after USMB treatment with scanning electron microscope (SEM, n = 3 at 0 day post USMB [0DPUSMB], 2 at 1DPUSMB, 1 at 1WPUSMB), US control without MB (SEM, *n* = 2 at 0 day post US (0DPUS), or transmission electron microscope (TEM, *n* = 3 0PUSMB); (2) AAV transfection efficiency with the cochleostomy and USMB‐RWM approaches (*n* = 5 per group); and (3) two control groups AAV transfection via RWM after (1) the ultrasound treatment without MB and (2) the treatment with MB but no ultrasound (n = 3 per group).

To evaluate the structural changes in the RWM by USMB, the middle ear was filled with the fixative immediately after ultrasound treatment to fixed the RWM. The cochlea was further fixed after the animal was sacrificed. To evaluate AAV transfection, the animals were subjected to a repeated ABR after a 2‐week interval prior to sacrifice. The cochleae were harvested and treated, to investigate either the structure of the RWM or the transfection of AAV across the neuroepithelium. All the experimental procedures were approved by the Institutional Animal Care and Use Committee of the Shanghai Sixth People's Hospital affiliated to Shanghai Jiaotong University (permit number DWLL2017‐0295).

### 2.2 ABR recording

The animals were anesthetized via an intraperitoneal injection of ketamine and xylazine (40 and 10 mg/kg, respectively) and placed on a thermostatic heating pad to maintain the body temperature at ~38°C. The ABR tests were performed in an acoustically and electrically shielded room. The acoustic stimuli were generated, and the responses were recorded using TDT System III (Tucker–Davis Technologies, Alachua, USA). The acoustic stimuli were 10 ms tone bursts at 1, 2, 4, 8, 16, and 32 kHz (rise–fall time, 1 ms; delivery rate, 21.1/s). The acoustic signal was delivered from a broadband speaker (MF1, TDT) via a plastic tube inserted into the external ear canal for the test in closed field. The biological responses were detected using three subdermal electrodes inserted at the vertex for recording and both mastoid regions for reference and grounding. The responses were amplified 20×, bandpass filtered between 0.1 and 3 kHz and averaged up to 1,000 sweeps per trial. At each frequency, the sound level was initiated at 90 dB SPL and decreased in 5 dB steps until the threshold was reached, which was defined as the lowest stimulus level at which a visible and repeatable wave III could be identified.

### 2.3 Fabrication of the ultrasound probe and assembly of the ultrasound generator

**FIGURE 1:**
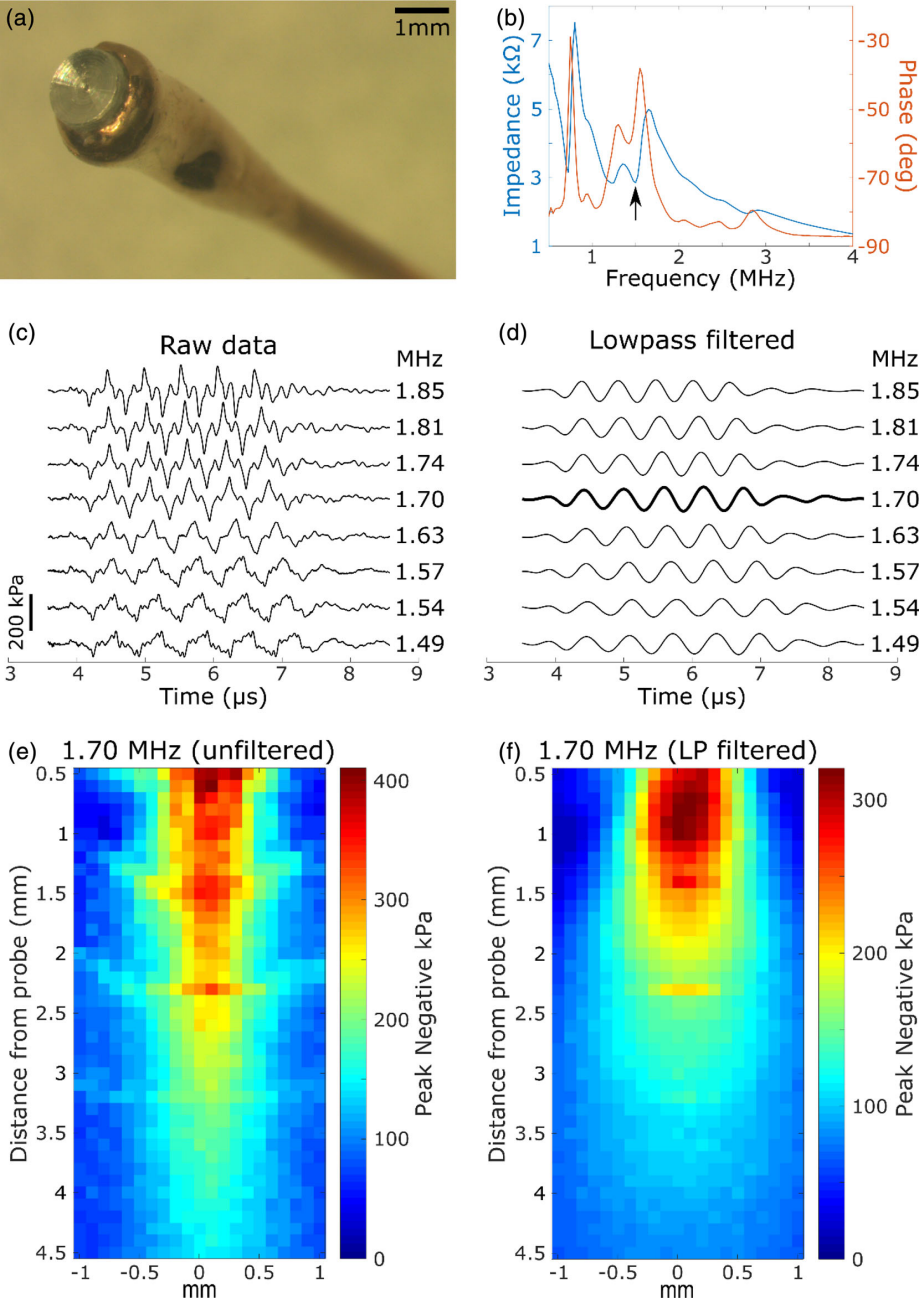
Ultrasound probe and acoustic measurements. (a) Photograph of the tip of the finished ultrasound probe, showing the aluminum lens and copper layer. (b) Impedance and phase response curves. The resonance peak at 1.55 MHz (indicated by the arrow, i.e., phase peak) was targeted for probe activation by the pulse pattern. (c) Raw acoustic pressure waveform at a fixed distance of 5.8 mm from the probe after electrical activation. This distance allowed the complete distinction of the acoustic response from the electrical stimulus artifact. The probe was stimulated with five cycles of ±30 V square waves at the indicated frequencies. (d) The data from (c) are presented after low‐pass filtering with a cutoff of 3.5 MHz. The greatest filtered amplitude was observed at 1.70 MHz, and this frequency was selected as the best MB cavitation frequency for the probe. (e) The peak negative pressure over a volume was recorded in response to a 1.70‐MHz stimulus. The maximum axial slice is shown. (f) The data from E are shown after low‐pass filtering of the waveform at each location as in (d) relative to (c)

The ultrasound transducer was constructed from a 1 mm thick piezoelectric substrate (PZT‐5A 1‐3 composite) with a 0.65 volume fraction (Smart Materials, Sarasota, FL). The material was cut into pieces of 1.5 mm diameter. A micro‐coaxial cable‐insulated core was fed through a hypodermic needle and connected to the back face of the transducer with E‐Solder 3022 conductive epoxy, and the cable shielding was connected to the needle shaft. Epo‐Tek 301 epoxy loaded with alumina was used to electrically isolate the back‐face connection and affix the transducer alignment with the needle axis. Next, a layer of copper was vacuum deposited (Mantis Deposition) over the face of the transducer and the side of the needle to create electrical continuity. An aluminum lens of the same diameter was machined with a geometrical focus of 2 mm and approximate height of 350 μm. The lens was attached to the transducer face with 301 epoxy. A SMA connector was attached to the free end of the cable (Figure 1a). The impedance/phase‐frequency curves (Figure 1b) exhibited multiple peaks, with a large resonance between 1.5 and 2 MHz, which is the expected from the thickness mode resonance for the 1 mm substrate.

US pulses were produced using a STHV748 pulse‐generating board (STMicroelectronics, Digi‐Key, Thief River Falls). The pulser board was programmed to generate 15 cycles of a negative‐leading biphasic square wave at a frequency approximately equivalent to the resonant frequency of the transducer. The pulse repetition period was 0.1 ms. Two variable output voltage supplies were connected to the board and adjusted to generate a signal range of up to ±90 V. The acoustic output was recorded using a needle hydrophone (Precision Acoustics, Dorchester, Dorset, UK) over a small volume (~10 mm^3^) in front of the lens to determine the spatial profile of the acoustic radiation (Figure 1c–f). The ultrasound waveform is presented as raw data (Figure 1c) and after low‐pass filtering at 3.5 MHz (Figure 1d), with respective peak negative pressures at 1.81 and 1.70 MHz. Those values were in the range of resonance between 1.5 and 6 MHz for the MBs with the diameters of 1–4 μm. The US peak negative pressure (tested under a driving voltage of 30 V and frequency of 1.7 MHz) varied between 0.4 and 0.35 MPa in the range of 0.5–1 mm from the front surface of the probe lens, yielding a variation in MI between 0.3 and 0.27.

**FIGURE 2:**
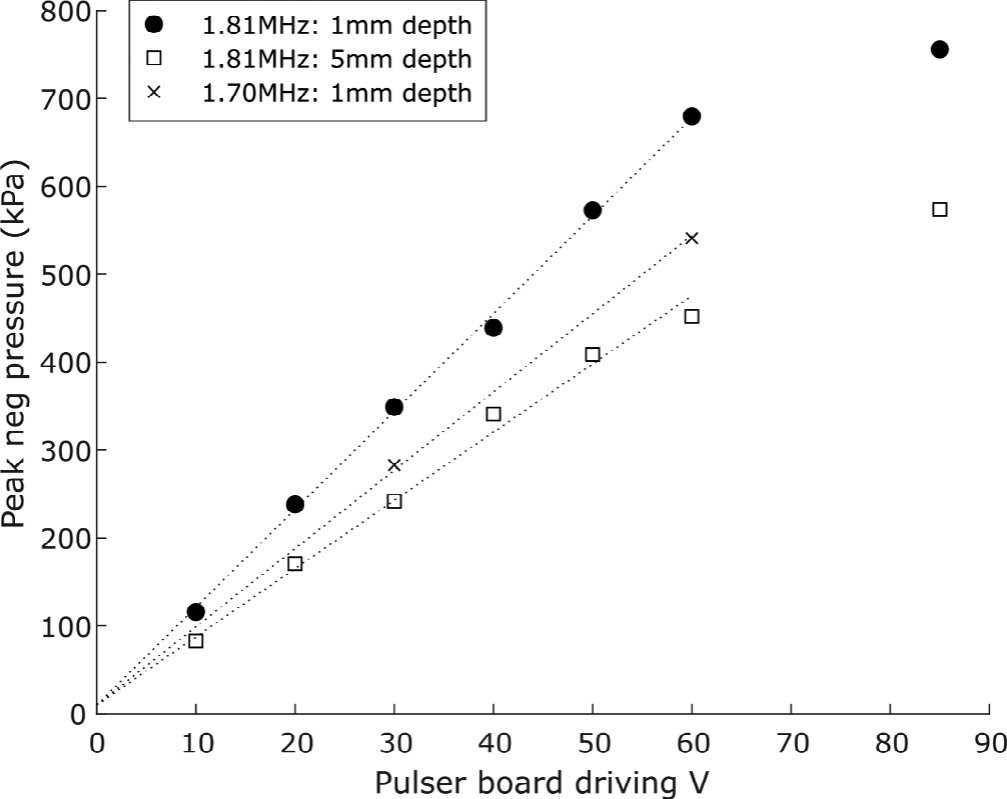
Peak negative pressures at fixed distances from the probe tip with varying pulser driving voltages. The distances and frequencies for each curve are given in the inserted legend. Dotted linear fit lines up to ±60 V are shown with the intercept fixed at the approximate noise floor of 10 kPa, demonstrating excellent output linearity. The pulser output began to saturate above 60 V (data not shown), and this is reflected in the measured pressure at 85 V, which was lower than predicted by the fit lines

Figure 2 shows that the peak negative pressure changes as a linear function of the pulse driving voltage up to 60 V. Based upon the MI obtained at a 30 V pulse driving voltage and 1.70 MHz and the linearity in the I/O function, 0.5 MI could be reached at a voltage of approximately 50 V at the distance of 0.5–1 mm. This voltage was used during the USMB treatment of the RWM.^38^

### 2.4 Surgery for gene transfection or RWM treatment

**FIGURE 3:**
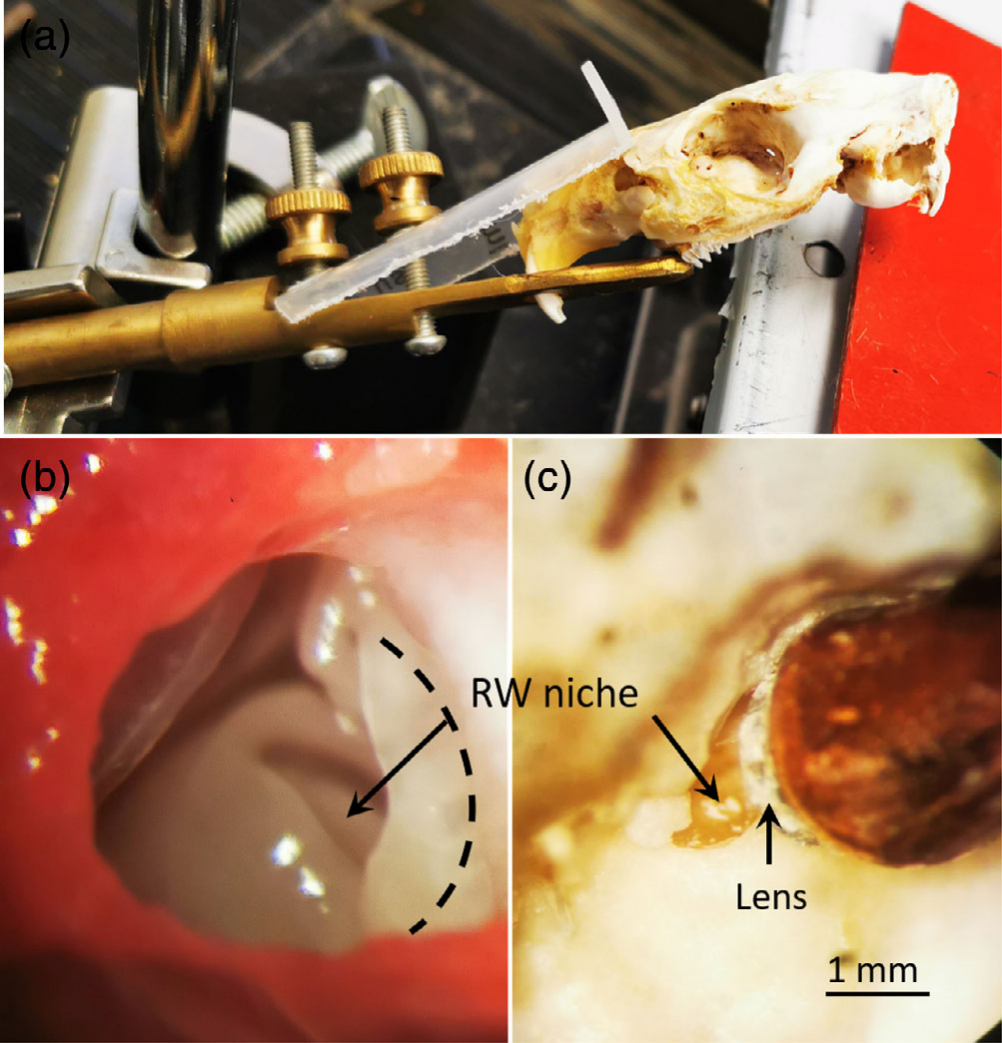
Photographic images of the USMB surgery. (a) The guinea pig skull was held in place using a homemade clamp in an orientation appropriate for the application of US probe. (b) A 3 mm hole was made on the mastoid to expose the RW niche. The dashed line indicates the edge to which the temporal bone would be trimmed. (c) The lateral view of the cochlea, with the ultrasound probe placed close to the RW niche. The probe was not fully inserted to allow a view of the RWM

The subjects were anesthetized with inhaled isoflurane (4% for induction, 2% for maintenance, 0.3 L/min O_2_ flow rate). The animal's head was placed in the lateral position and fixed with a stereotaxic restraint. The body temperature was maintained using a thermostatic heating pad at 38°C. The animal was laid laterally, and the head was held in position using a custom‐made holder (Figure 3a). The tympanic bony bulla was exposed using a post‐auricular approach. After administering local analgesia with lidocaine, a 2 cm arc incision was made along the root of the earlobe, and the mastoid was exposed via blunt dissection. A hole with a diameter of 3–4 mm was made on the bulla to expose the RW niche and the bony cochlear wall. Next, the animal was laid in the lateral supine position, and the head orientation was adjusted such that the RW surface faced up (Figure 3b). The ultrasound probe was inserted into the correct position against the RW niche with assistance from a manipulator. The lower edge of the probe front was placed on the RW niche (Figure 3c). The estimated distance between the front surface of the probe lens and the RW was 0.5–1 mm (Figure 3c).

For the USMB treatment of the RWM, the ultrasound contrast agent (Definity, USA, DIN:02243173) was prepared and injected into the RW niche to fill the space between the probe lens and RWM completely. The US generator was turned on to yield 5 min of sonication. After the US exposure, the MB solution was suctioned, and the middle ear cavity was irrigated with sterile saline and the residual solution was cleaned.

To observe the damage created by the USMB treatment, a fixative solution (2.5% glutaraldehyde) was used to fill the middle ear cavity immediately after washing. The animal was then sacrificed with an overdose of injected pentobarbital (100 mg/kg, i.p.). The animal was then decapitated under deep anesthesia, and the cochlea was harvested.

For AAV transfection via the RWM, a piece of gelfoam was placed in the RW niche after the US treatment. Ten microliters of an AAV solution were injected into the gelfoam. For AAV transfection via cochleostomy, a small hole (diameter: 0.3 mm) was drilled via the bone shell of the basal turn. Ten microliters of viral vector were injected into the scala tympani (rate: 20 nL/s) through a 34‐gauge glass tip (microfil) connected to a picrosyringe pump (Micro4; WPI, Kissimmee) by a polyethylene tube. The cochleostomy hole was then sealed with muscle tissue, and the hole of the bulla was closed by suturing the muscle and skin.

The adapted AAV2/Anc80L65 backbone was similar to the vector in a previous report.^36^, ^37^ The rAAV vector was constructed to carry an AAV2 ITR‐flanked genome encoding CAG‐driven eGFP, a Woodchuck Hepatitis Virus Regulatory Element (WPRE) and a bovine Growth Hormone poly‐adenylation site (Taitool Bioscience, China). The vector was presented at a titer of 1.16 × 10^13^.

### 2.5 Tissue preparation for morphology

After the MB solution was removed, the middle ear was filled with a fixative [4% paraformaldehyde in phosphate‐buffered saline (PBS)] before or after the cochlea was harvested (depending on the time points of the observation). Most of the temporal bone and the apical and the middle turns of the cochlea tissue were trimmed off. The remainder of the cochlea was then immersed in a solution containing 2.5% glutaraldehyde at 4°C overnight and post‐fixed in 2% OsO_4_ for 2 h. For SEM observation, the sample was dehydrated through a gradient series of ethanol concentrations (concluding with 100%) and treated with liquid CO_2_ for critical point drying. Before dehydration, the RWM was cut along the bony shell for approximately a quarter of the ring to reduce the tension by dehydration. Finally, the RWM was sputter‐coated with gold and observed using the SEM system (FEI Tecnai Spirit G2 Bio TWIN, Netherlands). For TEM observation, the RWM was cut down from the bony niche after fixation. The sample was dehydrated and embedded in Epon 812. Semi‐thick sections (70‐nm) were cut using an EMUC7 instrument (Leica) and observed using a TEM system (Tecnai G2 Spirit TWIN FEI, Brno, Czech Republic).

In the cochleae transfected with AAV‐eGFP, the basilar membranes were dissected from cochlea that had been fixed in 4% paraformaldehyde overnight at 4°C and then decalcified in 0.1 M ethylenediaminetetraacetic acid for 2 h. To distinguish hair cell transfection, the basilar membrane pieces were stained with phalloidin (Alexa Fluor 555‐phalloidin, cat. #8953S), mounted on microscopic slides, double‐stained with 4,6‐diamidino‐2‐phenylindole (DAPI; Fluoro‐shield with DAPI; Sigma‐Aldrich, cat. #F6057) and a coverslip was placed. After all staining procedures, the samples were examined using fluorescent microscopy (Axioplan2, Zeiss, Thornwood, NY) or confocal microscopy (LSM 710 META; Zeiss, Shanghai, China). For data processing, the percentage of eGFP‐positive HCs were manually quantified along the cochlea, by counting the number of eGFP‐positive, phalloidin and DAPI‐positive HCs, per 0.24 mm sections per basal sample for each specimen.

### 2.6 Statistics

All data are expressed as means ± SE of the mean (SEM). ANOVAs followed by post hoc testing (Holm‐Sidak method) were performed using SigmaPlot (ver. 14; Systat Software Inc., San Jose, CA). In all analyses, *p* < 0.05 was taken to indicate statistical significance. In all analyses, a *p* value <0.05 was considered to indicate statistical significance.

## 3 RESULTS

### 3.1 The RWM damage induced by USMB cavitation

**FIGURE 4:**
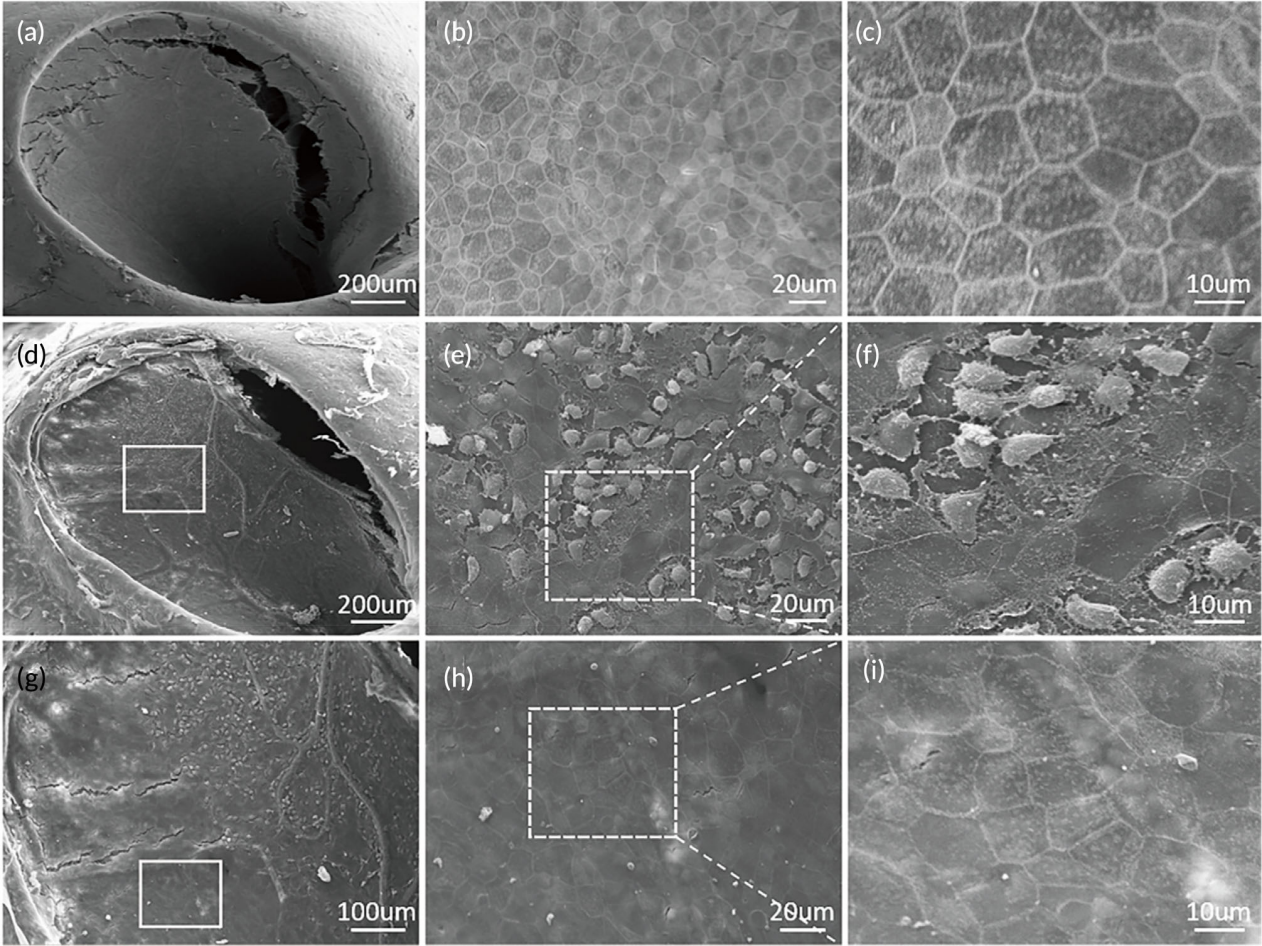
Scanning electron microscopy (SEM) view of the RWM surface facing the tympanic cavity. (a) The whole RWM view of a control sample. (b and c) High‐magnitude views of the control. (d) The whole RWM view of a USMB treated sample; the white square is in the damaged area that is magnified in (e) and (f). (g) The X2 view of D for a better view on the board between the damaged and undamaged areas; the square in (g) depicts the undamaged region that is magnified in (h) and (i)

The RWM damage induced by USMB cavitation was observed in both SEM and TEM images. In the SEM images of the control RWM (Figure 4a–c), the epithelial surface was clean, and the cells had a square‐shaped appearance and tight adhesions to their neighbor cells. In the RWM observed immediately after the USMB treatment, a focused region could be identified as being damaged, while the other regions appeared to be normal. The damaged region took approximately 1/3 of the total RWM area and located anteriorly. A square region was circulated in Figure 4d, which was magnified in E and F to show the detail of the damaged epithelial layer. The damaged cells frequently contained round‐shaped, scar‐like structures, which were likely the residuals of large MBs. Figure 4g is also a magnified image of Figure 4d, which allows a better view of the border between damaged and undamaged region. A square area of undamaged surface was magnified in Figure 4h,I as a good self‐control with no damage on RWM surface.

**FIGURE 5:**
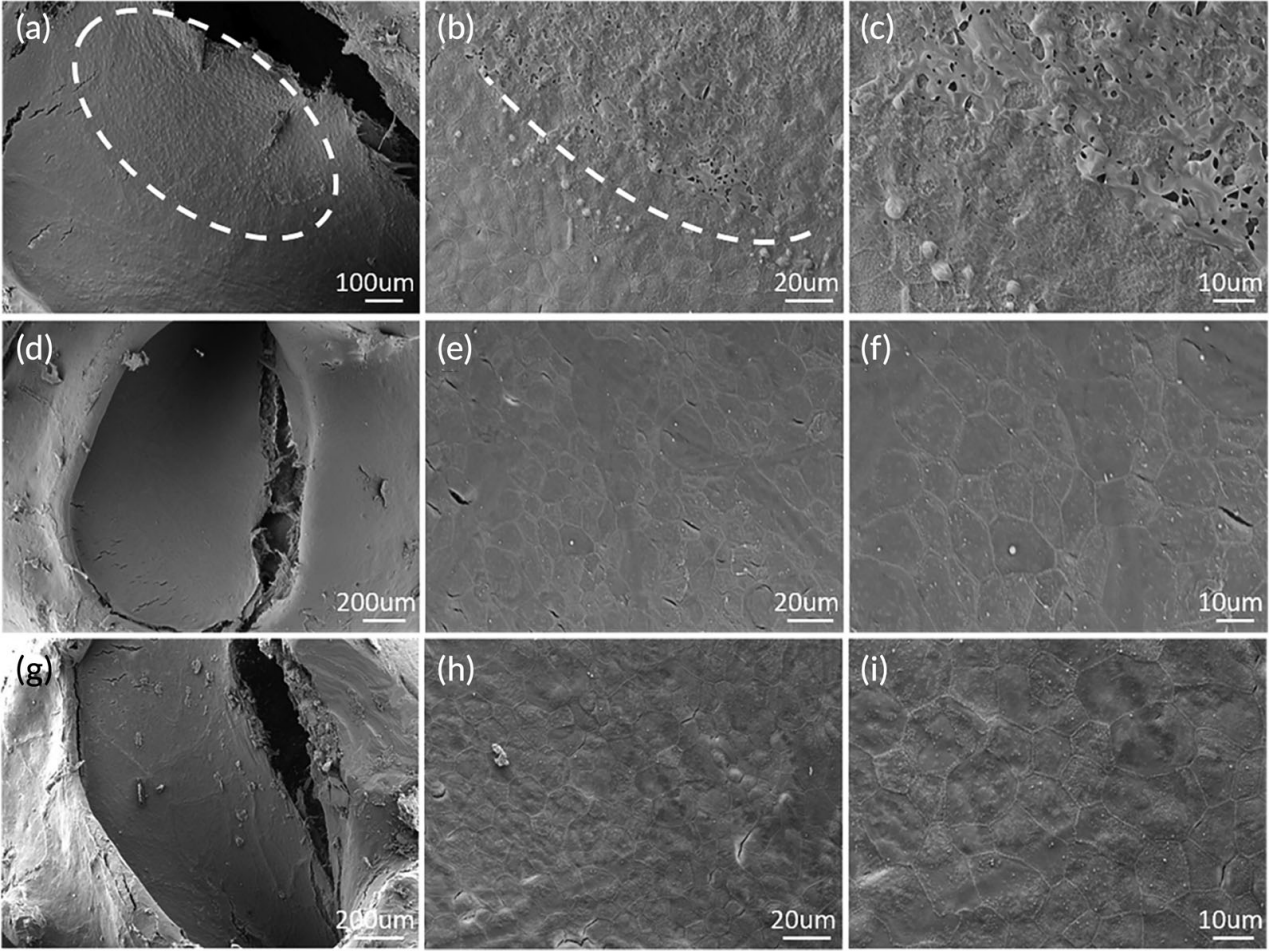
SEM images of the RWM observed at different time points after USMB treatment and the RWM with ultrasound‐only treatment. (a)–(c) 1 day post USMB treatment. The damaged region was circled in (a) and bordered in B by dashed line. (d)–(f) RWM 1 week post USMB treatment. (g–i) RWM observed immediately after ultrasound‐only treatment

To observe the recovery of RWM after the USMB damage, RWM samples were observed at 1 day and 1 week post the USMB (1DPUSMB and 1WPUSMB). At 1DPUSMB, the RWM damage was not fully healed (Figure 5a–c for different magnifications). The damaged area was covered with a capsular structure, which may be the crawling of new cells that were repairing the damaged epithelial surface (Figure 5c). Again, the damage region was focused in the 1/3 area located anteriorly as circulated by the dashed oval. The dashed line in Figure 5b defined the boarder between damaged and undamaged region. The sample observed 1WPUSMB shows no difference from the untreated control, suggesting a full recovery of the wound (Figure 5d–f). Figure 5 also showed the image of a control sample observed immediately in which RWM was only exposed to ultrasound but no microbubbles (Figure 5g–i), the epithelial layer of RWM was nearly intact in those images.

**FIGURE 6:**
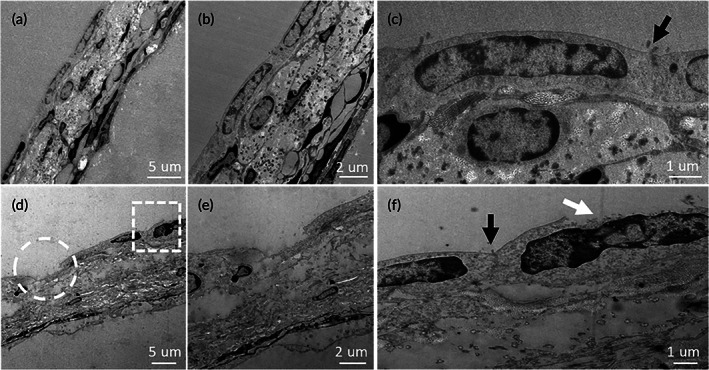
TEM view of the RWM. (a–c) Normal RWM structure. (d–f) RWM damage induced by USMB treatment. The black arrows in (c) and (f) point to the tight junctions seen at the outer surface. The circle in (d) identifies the region magnified in (e) to demonstrate that the outer epithelial cells are totally disrupted to the middle layer of the RWM. The square in (d) identifies a region where the damage is limited to the outer surface of the epithelial cell (magnified under the white arrow in (f))

Figure 6 presents the TEM images to show the structures of RWM across different layers. The images of the normal control (A, B and C) show a sandwich structure in which two layers of epithelial cells were separated by a layer of continuous tissue. All three layers demonstrated a good continuity, and good tight‐junction between cells in the outer epithelial. In the USMB treated RWM (Figure 6d–f) however, the continuity of the outer epithelial was interrupted (as shown in the white cycle in Figure 6d). This interruption of intercellular continuity was not extended to the deeper layers.

### 3.2 ABR threshold

**FIGURE 7:**
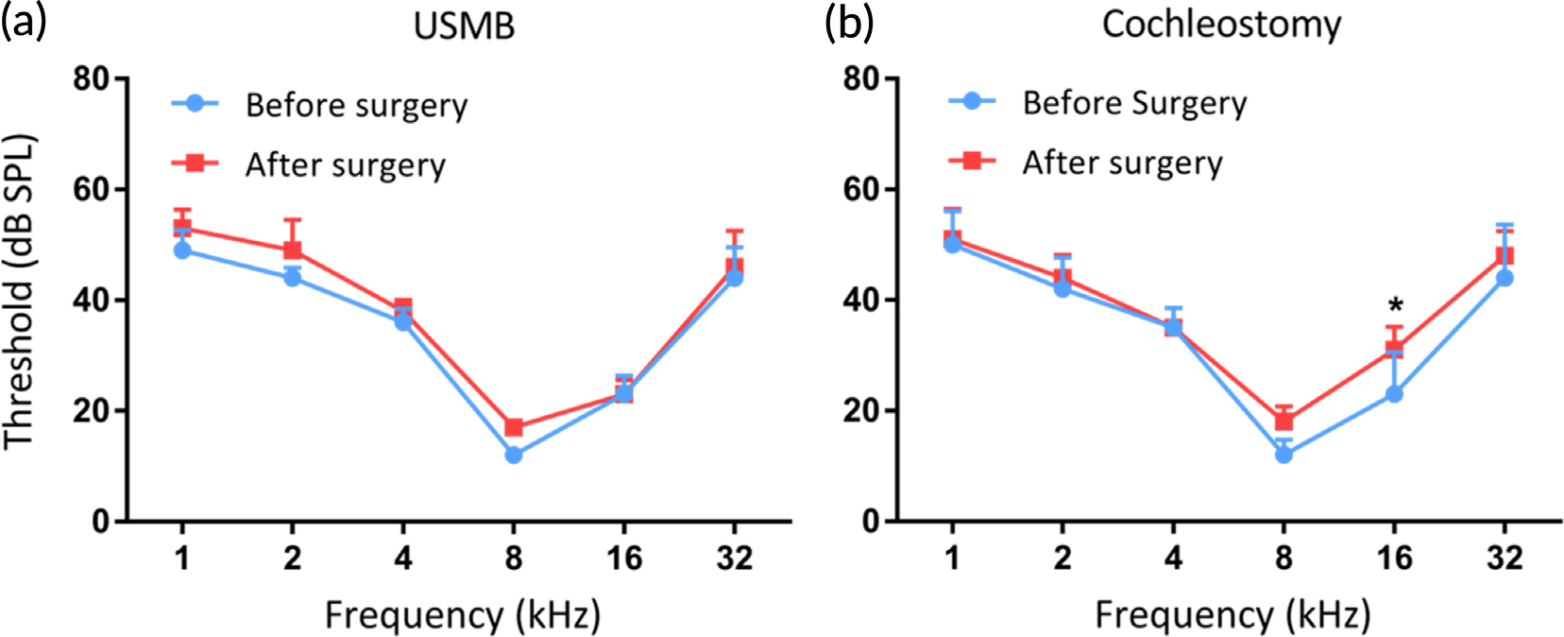
Tone‐burst ABR audiograms before and 2 weeks after the gene transfection surgery. The thresholds are the group means (±1 SEM) calculated for five ears per group

ABRs were tested to examine the hearing threshold at the baseline (i.e., before surgery) and 2 weeks after the transfection surgery. The results in Figure 7 show that AAV delivery via the RWM after USMB treatment does not cause a shift in the ABR threshold (Figure 7a). In contrast, a small ABR threshold elevation was observed in subjects treated with cochleostomy, in which the post hoc pairwise test revealed a significant threshold shift at 16 kHz (Figure 7b) relative to the baseline (*q* = 3.336, *p* = 0.023). A significant between‐group difference was revealed by a two‐way ANOVA (*F*_1,48_ = 6.391, *p* = 0.015).

### 3.3 Cochlear AAV transfection

**FIGURE 8:**
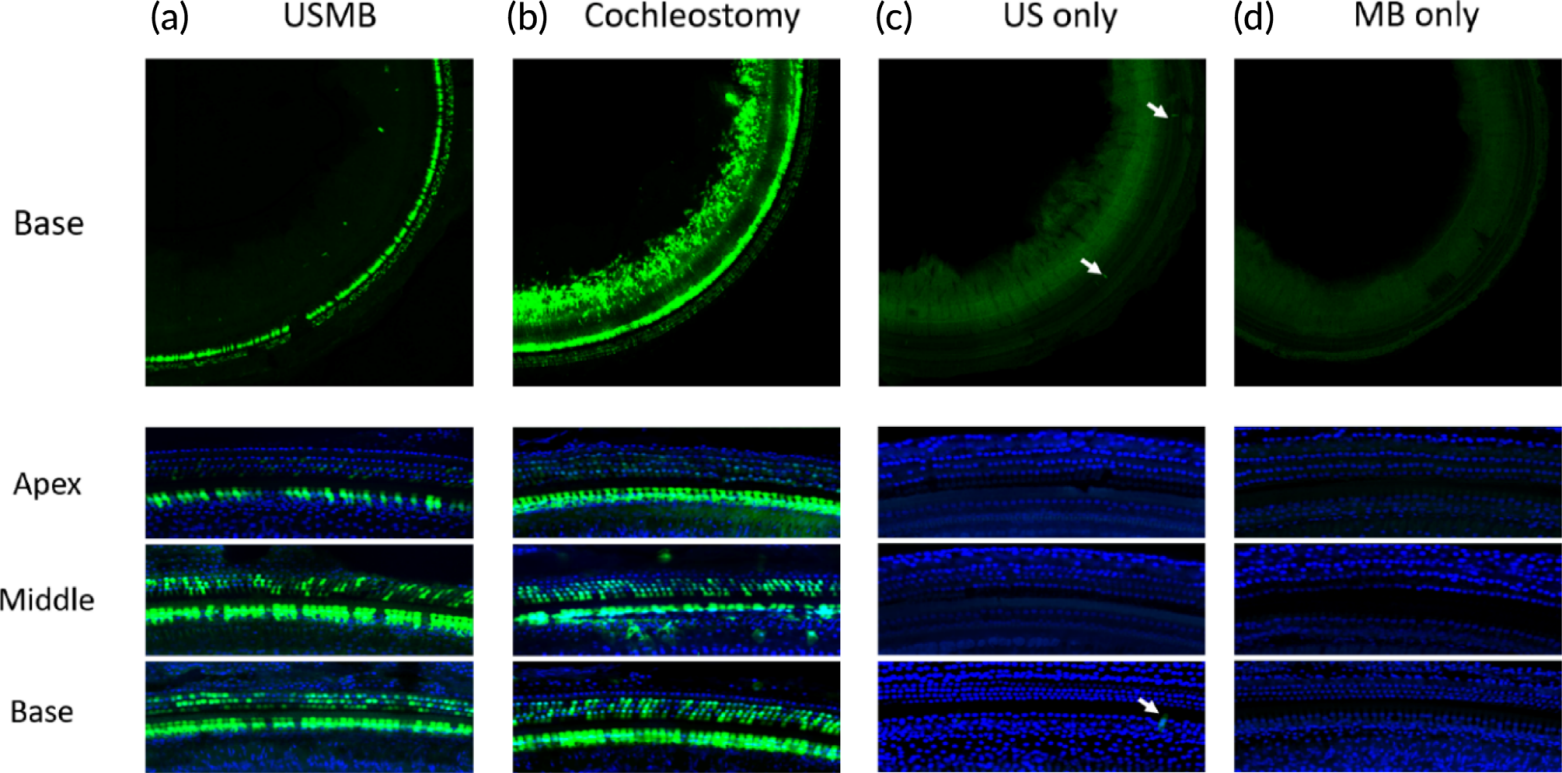
Typical confocal images of eGFP expression following transfection with 10ul AAV2/Anc80L65 in cochlear surface preparations. (a) Transfection via the RWM with USMB treatment. (b) Transfection via cochleostomy. (c) Transfection via the RWM treated with US only. (d) Transfection via the RWM treated with MB only. A satisfactory but slightly weaker level of transfection was observed in the USMB‐treated cochleae relative to the cochleostomy‐treated cochleae. Virtually no effective transfection was observed in the two control samples (US only and MB only) other than a few scattered eGFP‐positive cells in the US‐treated sample

Figure 8 presents typical images of eGFP expression in the prepared cochlear surfaces of different groups. Overall, transfection with cochleostomy (Figure 8b) yielded a higher level of expression than that accomplished with the USMB method (Figure 8a). Under the two control conditions (C for US only and D for MB only), virtually no effective transfection was observed throughout the cochlea.

**FIGURE 9:**
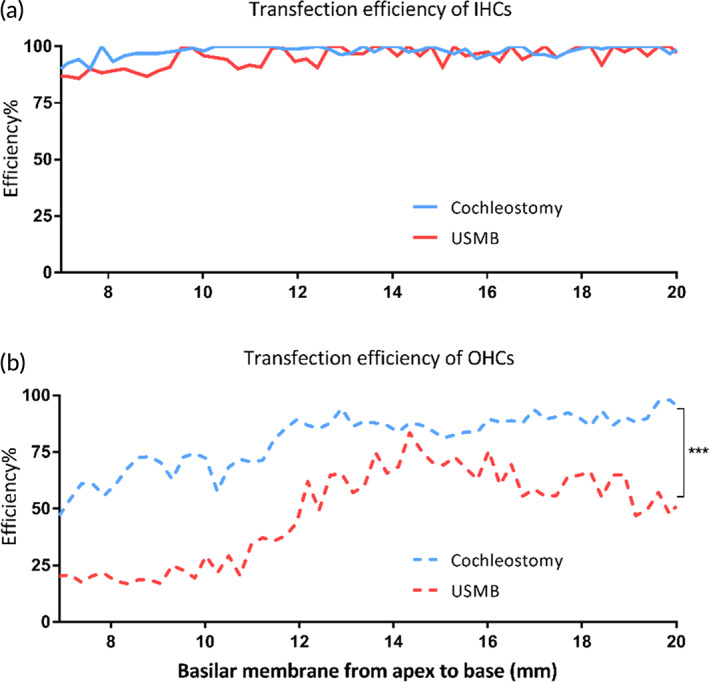
The averaged cochleograms (*n* = 5) for the percentages of IHCs (a) and OHCs (b) achieved via cochleostomy or USMB

Figure 9 shows the transfection cochleograms for both IHCs (A) and OHCs (B). The transfection efficiencies in IHCs at the basal turn were 98.4 ± 1.7% and 97.2 ± 3.0% for both cochleostomy and RWM approach respectively. The difference between the two methods was not significant (*n* = 5 in each group, *t* = 0.938, *p* = 0.376). The corresponding transfection rate of OHCs was 89.4 ± 4.3% in cochleostomy, which is significantly higher than the value of 62.5 ± 8.8% in RWM approach (*t* = 10.313, *p* < 0.001).

## 4 DISCUSSION

### 4.1 Short summary

In this study, we used a homemade ultrasound probe with a transducer diameter as small as 1.5 mm. When the probe was inserted against the RWM niche, we managed to maintain the structural integrity of middle ears necessary for good hearing. USMB‐mediated cavitation caused controllable, focused, and reversible RWM damage (Figures 4 and 5) that was limited to the outer epithelial layer (Figure 6). This treatment effectively increased the permeability of RWM to the rAAV, which could not normally pass across the RWM to transfect cochlear cells (Figure 8c,d), resulting in the satisfactory transfection of cochlear sensory cells (Figure 8a and 9) by using AAV2/Anc80L65. Although the RWM approach yielded a lower transfection rate than cochleostomy, the former approach did not affect the hearing thresholds of treated animals.

### 4.2 The advantage of using small probe

The small probe size allowed us to insert the probe in touch with the RWM niche in the guinea pig ear. Therefore, the estimated distance between the probe lens and the RWM was within 0.5–1 mm (Figure 3c). At this distance, our tests indicated that the peak negative acoustic pressure delivered by our probe typically reached 0.3–0.5 MPa, and MI adjusted to 0.5. Placing the probe against the RW niche enabled a much better focus on the target and required a reduced device output, as much less energy was lost to attenuation. The focalized damage to RWM was shown in both Figures 4d and 5a. In fact, in all the sample treated with USMB, the damage was limited to an oval shape region in the anterior 1/3 of RWM. This is well corresponding to the pointing direction of our probe as shown in Figure 3c. Meanwhile, the focus of the US energy on a small spot minimizes the risk of collateral damage to non‐target tissues. Another advantage of using a small probe involves the reduced amount of MB solution. Because the probe is placed directly against the RW niche, a small amount of solution would be enough to fill the space between the probe lens and RWM to create an no‐air contact, in contrast to the filling of the whole middle ear cavity if a larger probe is used. This may be beneficial for the co‐administration of the MB solution with rAAV. The use of small probe increases the feasibility of this technique to be translated into the cochlear gene therapy in human subjects, especially for the purpose of hearing protection in subjects without severe hearing loss. The small probe size allows the probe to be inserted via eardrum with a repairable perforation. While using a commercial probe of 6 mm diameter, the probe must be place in the external ear canal, far away from RWM. In this case, the middle ear must be filled fully with microbubble solution.

### 4.3 USMB methods for cochlear drug delivery

Collectively, only two studies published by one group have addressed the use of USMB in drug delivery across the RWM.^29^, ^34^ In these reports, the ultrasound probe diameter was 6 mm. Moreover, the probe was placed outside the middle ear, which must then be filled fully with MB solution. The estimated distance between the front surface of the probe and the RWM was 5 mm. The acoustic intensity was 1–3 W/cm^2^, which corresponded to a MI of 0.147–0.283. In this setting, USMB considerably enhanced the transportation of biotin‐FITC, which can permeate the intact RWM. The integrity of the RWM in response to this USMB treatment method was reported more recently in a separate paper by the same group.^35^

### 4.4 USMB in rAAV‐mediated cochlear gene therapy

US has long been recognized as a useful tool for targeting material delivery for therapeutic applications, including gene transfection (see reviews^26^, ^31^, ^32^, ^39^, ^40^, ^41^). MBs have been used as imaging enhancers since the 1990s.^41^, ^42^ Shortly thereafter, the application of MBs was extended to therapeutic areas.^43^, ^44^

Several potential mechanisms have been proposed to explain how USMB methods enhance cell permeability and drug uptake. Depending on the magnitude of the US driving pressure, the MB response may shift from linear spherical to nonlinear or nonspherical oscillations and eventually to inertial cavitation.^31^ At a driving pressure greater than 300 kPa, the fluid inertia will overcome the pressure inside the MBs, resulting in bubble collapse and/or fragmentation.^45^, ^46^ The surrounding cells exposed to the shock waves and jet formation associated with cavitation can incur damage ranging from small and temporary pores (~1 μm in diameter), which heal quickly,^30^, ^47^, ^48^, ^49^, ^50^ to large damage (>10 μm) associated with cell death.^49^, ^51^ A high‐speed camera revealed the formation of these small pores on the cell membranes in a microsecond time frame after a single US pulse, and the resealing of the pores within several seconds.^30^, ^52^, ^53^, ^54^ Such short‐lived damage is desirable for the delivery of drugs incorporated into or co‐administrated with MBs,^55^, ^56^ but may not be desirable for the AAV mediated transfection in which AAV is applied after USMB treatment.

In most USMB applications, the MBs must be applied to the space around the target cells because MB‐mediated cavitation affects only the cells within close proximity. As the cochlea is an isolated and structurally complex organ, it would be impractical to deliver MBs to the solution compartment around the target cells without disturbing the cochlear function. Moreover, the cochleae are surrounded by a bony shell that can largely attenuate the US. All these facts limit the use of USMB for cochlear gene transfection.

Because the target of gene transfection is the cells in the organ of Corti and, occasionally, the stria vascularis rather than the RWM, small pores that reseal within seconds are not adequate for gene transfection via a viral vector. These pores may ensure the uptake of virus by RWM cells but may not permit virus to cross the RWM and reach the target cells. Moreover, the high costs of rAAV vectors limit the coadministration of the vector with MB solution. Therefore, a large, long‐lasting, but healable wound must be created in the RWM to ensure the successful use of rAAV in cochlear gene transfection. In this study, focused ultrasound was used to create large areas of damage on the surfaces of several epithelial cells (Figure 4). The healing of such large wounds involves the proliferation of new cells and would require a considerably longer period as shown by our result (Figure 5). This would be desirable for the cross‐RWM transportation of rAAV after USMB treatment.

### 4.5 MB selection and RWM damage

MBs typically have diameters of 1–10 μm and comprise a gas core and lipid shell. For clinical applications, several features of MBs, such as high biodegradability, low immunogenicity, sufficient flexibility and stability, are of concern. Regarding cochlear gene transfection, the ability of MB to create RWM damage that would be sufficiently persistent but healable is the major concern. MB properties such as the shell material, size, and concentration are important because each may affect the induction of inertial cavitation under ultrasonic exposure.^57^, ^58^

Lipids, proteins, polymers, or a combination of these materials have all been used in the shells of MBs. MBs coated with lipids are among the most interesting and frequently used formulations in studies associated with drug delivery.^59^, ^60^, ^61^, ^62^, ^63^, ^64^ The MB cavitation effect appears to be related to the size and total volume of MBs in the solution. One study reported that both the inertial cavitation dose and the BBB opening volume were positively correlated with the diameters of the MBs.^65^ However, another study reported that the microbubble gas volume dose, not the size, determined the effect.^61^ This result was validated by Liao et al. when detecting cavitation in vitro.^66^

Definity® (Lantheus Medical Imaging), a commercial lipid‐coated MB agent, was used in the present study. Once fully activated by shaking (VIALMIX®), this product contains a maximum of 1.2 × 10^10^ perflutren lipid microspheres/mL, which is considerably higher than that of other products such as Sonovue® (Bracco Diagnostics; 1–5 × 10^8^). Up to 98% of Definity MBs have a diameter of <10 μm and a mean size of 1.1–3.3 μm. This size range is close to the 3.5 μm diameter MBs with a resonance that matches the probe resonance of approximately 1.7 MHz. Even if 3.5 μm exceeds the mean diameter for a given experiment, high numbers of 3.5 μm MBs should still be present.

### 4.6 Safety concerns

In this study, the intense damage caused by the application of USMB was limited to the RWM epithelial cells facing the tympanic cavity but was not extended to the deeper layers. In one of our previous reports, the RWM could be damaged using digestive enzymes.^24^ In this study, the damage was also limited to the outer epithelial layer. Functionally, we observed no hearing losses in subjects treated with either RWM digestion in the previous study or with USMB in the present study. These results suggest that the application of USMB to the RWM is a safe method for cochlear gene transfection. Moreover, the USMB method is more controllable than our previously reported digestion method.

Other than RWM approach, cochleostomy and canalostomy have been evaluated for cochlear gene transduction by AAV. In the best scenario, cochleostomy can achieve a safe cochlear gene transfection mediated AAV in large animal model like guinea pig with less than 10 dB threshold shift. However, such a good hearing reservation is difficult to be reached in adult mice in cochleostomy.^15^ AAV injection via canalostomy can effectively infect cells of cochleae and vestibular organs in neonatal mice without significant hearing loss.^11^, ^14^ However, the great recovery ability of neonatal mouse cochlea after intense surgical injury is not likely duplicable in adult mice. More importantly, both canalostomy and cochleostomy are less likely to be translated in human cochlear gene therapy, especially for the protection purpose in which hearing reservation is critical. Unlike rodents, human inner ears are deeply embedded in the temporal bone. Both cochleostomy and canalostomy require intense surgery, and likely risky in causing hearing loss. In humans, RWM approach is the only one that has been utilized for inner ear drug delivery (e.g., in the treatment of sudden sensorineural hearing loss and Meniere's disease^67^, ^68^).

### 4.7 Limitations and future improvements

In this study, we compared the transfection efficiencies between the USMB‐RWM approach and the cochlear injection of virus via cochleostomy. A slight hearing loss was observed in the subjects after cochleostomy but not after USMB. However, the transfection rate was significantly lower in the USMB group than in the cochleostomy group (Figure 9). While our focus is on cochlear gene transfection in this study, RWM approach is likely useful for gene transfection in the vestibular system, considering the fact that RWM is closer to vestibule than cochlea. We intend to evaluate this potential in our further study especially after the delivery system is optimized. Several possibilities for further improvements are under consideration. The first possibility involves the use of a smaller probe. Although the 1.5 mm probe allowed us to place the probe on the ring of the round window niche, the surgery required to open the area for access remains quite invasive. The reduction of the probe size to 1 mm would enable the surgery to be performed more easily, and the probe could be inserted into the niche along with a smaller amount of MB solution. However, the difficulty of manufacturing these devices increases as the diameter decreases. The second improvement involves the packaging of the rAAVs into MBs or the coadministration of rAAVs with the MB solution. A reduced probe size would make this approach possible. The third improvement involves the use of recently reported novel AAVs that have a higher transfection rate.^11^, ^12^, ^13^, ^14^, ^16^, ^69^, ^70^ We believe that with these improvements, USMB‐mediated cochlear gene transfection via the RWM would become a useful tool that could be translated into human clinical applications.

## 5 CONFLICT OF INTEREST

The authors declare no conflict of interest.

### PEER REVIEW

The peer review history for this article is available at https://publons.com/publon/10.1002/btm2.10189.
